# Lateral Hypothalamic Orexin Neurons Mediate the Reward Effects of Pain Relief Induced by Electroacupuncture

**DOI:** 10.3389/fnmol.2022.812035

**Published:** 2022-03-01

**Authors:** Can Wang, Meiyu Chen, Chuan Qin, Xiaoyi Qu, Xueyong Shen, Sheng Liu

**Affiliations:** School of Acupuncture-Moxibustion and Tuina, Shanghai University of Traditional Chinese Medicine, Shanghai, China

**Keywords:** acupuncture, reward, pain relief, orexin, lateral hypothalamus

## Abstract

The reward of pain relief caused by acupuncture has been found to be clinically significant. However, the molecular mechanisms underlying acupuncture-induced reward of pain relief in chronic pain remain unclear and have not been analyzed in suitable preclinical models. Here, we investigated whether acupuncture could potentially induce the reward of pain relief and orexin neuronal signaling in the lateral hypothalamus (LH) and exhibit a possible role in electroacupuncture (EA)-induced reward in spared nerve injury (SNI) rats. Therefore, by using conditioned place preference (CPP) paradigm, we noticed that EA induced the preference for cues associated with EA-induced pain relief in the early, but not late, phase of chronic pain. These observations were different from the immediate antihyperalgesic effects of EA. c-Fos/orexin double labeling revealed that EA stimulation on 14 days but not on 28 days after SNI modeling activated greater numbers of c-Fos positive orexin neurons in the LH after the CPP test. Moreover, the administration of an orexin-A antagonist in the LH significantly blocked the reward effects of pain relief induced by EA. Furthermore, by using cholera toxin b subunit combined with c-Fos detection, we found that the orexin circuit from the LH to the nucleus accumbens (NAc) shell was significantly activated after EA induced CPP. Microinjection of the orexin antagonist into the NAc shell substantially attenuated the CPP induced by EA. Intravenous injection of low-dose orexin-A together with EA resulted in significantly greater antihyperalgesia effects and CPP scores. Together, these findings clearly demonstrated that LH orexin signaling could potentially play a critical role in the reward effects of pain relief induced by acupuncture. The observations of the present study extended our understanding of orexin signaling in the LH and its role in EA-induced reward, providing new insights into the mechanisms of acupuncture analgesia.

## Introduction

It is well established that the current conceptualizations of pain in humans are predominantly multidimensional, mainly including the perception of the noxious stimulus and the affective features of pain. Moreover, both onset of pain and pain relief have been reported to be motivationally salient events, which can lead to generating avoidance (withdrawal) and approach behavior (Porreca and Navratilova, 2017). It is generally accepted that the relief of pain can give rise to negative reinforcement, which has been appropriately described as a reward (Navratilova and Porreca, 2014). The reward effects of pain relief could facilitate learning and memory associated with relief, and drive motivation and behavior to seek a cue (context) associated with pain relief that can significantly accelerate recovery. Mounting evidence also suggest that extensive brain areas [such as anterior cingulated cortex, hypothalamus, ventral tegmental area, and nucleus accumbens (NAc)] and neurochemicals (dopamine and opioids) can possibly overlap between pain and reward pathways (Watanabe and Narita, 2018; Serafini et al., 2020). For instance, reduced analgesic efficacy has been found to be related to maladaptation of the neural circuits involved in reward induced by pain relief (Serafini et al., 2020). In laboratory animals, the conditioned place preference (CPP) paradigm has been widely used to evaluate the reward effects of pain relief (Truini et al., 2013) and the efficacy of various analgesics, especially in alleviating the aversive aspects of pain (King et al., 2009; Navratilova and Porreca, 2014; Navratilova et al., 2015). Furthermore, investigations using the CPP paradigm in rats have been found to be consistent with psychological studies in humans that can conceptualize relief of pain as a reward (Leknes et al., 2008; King et al., 2009).

Accumulating evidence has demonstrated the effectiveness of acupuncture in the clinical treatment of both acute and chronic pain (Vickers and Linde, 2014; Vickers et al., 2018). Acupuncture or electroacupuncture (EA) can not only alleviate the sensory of the noxious stimulus, but can also markedly inhibit pain affect (Zhang et al., 2012; Duanmu et al., 2017). However, the reward effect of pain relief induced by acupuncture has not been well studied in preclinical models. In fact, various lines of evidence suggest that acupuncture-induced reward of pain relief can be clinically significant. First, many patients with different types of pain feel pleasure after receiving acupuncture treatment and an expectation of pain relief can produce markedly greater acupuncture analgesia in patients with pain (Kong et al., 2009). Second, it has been shown that the cognitive and emotional control of pain is modulated effectively by different reward/motivational circuits (Elman and Borsook, 2016). Interestingly, fMRI studies have demonstrated specific functional responses in the brain reward systems associated with acupuncture analgesia (Wang Z. et al., 2017; Yu et al., 2020b). Third, altered neuronal processing in the reward/motivational systems may also be involved in the regulation of pain sensitivity and tolerance (DosSantos et al., 2017; Yu et al., 2020a). This raises an important issue that should be addressed in preclinical models, namely, whether the pain relief produced by acupuncture can possibly induce a reward and drive pain relief-related approach behaviors that can potentially accelerate the recovery. Such findings may greatly help to extend our understanding of the mechanisms underlying acupuncture analgesia. Given that chronic pain can induce long-term plasticity in the brain, and neuroplasticity can adversely affect pain behaviors ranging from symptomology to the treatment response in different stages of pain (Latremoliere and Woolf, 2009), we aimed to assess the CPP induced by acupuncture during different durations of chronic pain by using a spared nerve injury (SNI) model.

Orexin (also known as hypocretin) specifically located in the hypothalamus is produced from a prepro orexin molecule orexin neurons. Several lines of evidence have suggested that the orexin neurons in the lateral hypothalamus (LH) may play a pivotal role in reward effects, including drug, food, and alcohol rewards (Harris et al., 2005; Harris and Aston-Jones, 2006). LH orexin neurons have been found to be related to behavioral preference with c-Fos activation during both the food and drugs reward processing (Harris et al., 2005; Marchant et al., 2009). Notably, the orexin system has also been shown to be involved in analgesia and has been implicated in the descending pain inhibition (Ossipov et al., 2010). Activation of orexin neurons can give rise to analgesic effects in both neuropathic and inflammatory pain conditions and can effectively suppress the aversive response to various noxious stimuli (Zhou et al., 2018). Interestingly, several studies have suggested that acupuncture analgesia might involve the orexin neuronal system. For instance, Feng et al. (2012) found that EA could significantly alleviate postlaparotomy pain through the spinal orexin-A receptor. Recent work has also shown that the median nerve electrical stimulation (EA at SP6) can induce the activation of hypothalamic orexin neurons *via* the activation of cannabinoid receptor 1, and can thereby mediate the disinhibition of the periaqueductal gray (PAG), which can effectively contribute to EA analgesia (Chen et al., 2018). In addition, there is extensive innervation of the NAc by LH orexin neurons, and orexin receptors have also been found to be highly expressed in these areas (Patyal et al., 2012; Burdakov, 2020; Khosrowabadi et al., 2020). The NAc can integrate the rewarding valence of the stimuli and exhibit plastic adaptations in pain processing (Massaly et al., 2019; Makary et al., 2020). We hypothesized that LH orexin neurons and their potential neural circuit to the NAc represent an important mechanism for regulating acupuncture-induced reward of pain relief.

## Materials and Methods

### Animals

Adult male Sprague-Dawley rats (230–300 g; Slack) were purchased from the Laboratory Animal Center of Shanghai University of TCM. The rats were allowed to acclimatize for 7 days. The rats were housed in a clean animal environment (temperature of 24 C ± 2 C, 12/12-h light/dark schedule). The animal experiments were approved by the Shanghai University of TCM Animal Ethics Committee (SZY 201710008). The animal license was No. PZSHUTCM210709001. The experiments were conducted in accordance with NIH standard guidelines for the use and care of experimental animals.

### Drugs

Orexin-A (purchased from Tocris Bioscience, Minneapolis, MN, United States) was dissolved in 0.9% sterile saline. The orexin-A receptor antagonist SB-334867 (SB; obtained from MedChem Express, Monmouth Junction, NJ, United States) was dissolved in a vehicle [50% dimethyl sulfoxide (DMSO), 50% saline] to prepare a stock solution of 3.0 nmol/0.3 μl for use. The dose was selected according to a previous study (Narita et al., 2006). Cholera toxin b subunit [CTb; Sigma, St Louis, MO, United States, 0.5% dissolved in 0.1 M phosphate buffer (PB)] was used for retrograde neuronal tracing.

### Spared Nerve Injury Animal Model

As described in the previous studies (Decosterd and Woolf, 2000; Shields et al., 2003), the rats were anesthetized with isoflurane (5%). To separate the biceps femoris and to expose the sciatic nerve and its associated branches, an incision proximal to the lateral side of the right knee was made and thereafter the biceps femoris was separated. The sciatic nerve and its branches were exposed. A silk suture was then used to ligate the common peroneal and tibial nerve branches. We removed about 1 mm of the nerve and kept the sural nerve intact. In the Sham SNI group, the operation was performed by following the same procedure, but we did not cut-off or ligate the sciatic nerve, peroneal nerve, and tibial nerve branches.

### Electroacupuncture Treatment

We first restrained the rats gently. Thereafter, the rats were subjected to EA with two stainless steel needles (0.3 mm diameter) inserted bilaterally into ST36 and SP6 at a depth of 5 mm (Figure 2A), as previously described (Wang X. et al., 2017). Acupuncture point ST36 is located between the muscle anterior tibialis and muscle extensor digitorum longus near the knee joint. In contrast, SP6 is located at 3 mm proximal to the superior border of the medial malleolus. A constant current square wave electric stimulation produced by using a stimulator (Model G-6805-2, China) was continuously transferred *via* the needles. We set a 2-Hz stimulation frequency with increasing stimulation intensity (from 0.5, 1.5 to 2 mA, 10 min each step). In the Sham EA, the needles were inserted into acupoints, but without any electric stimulation.

**FIGURE 1:**
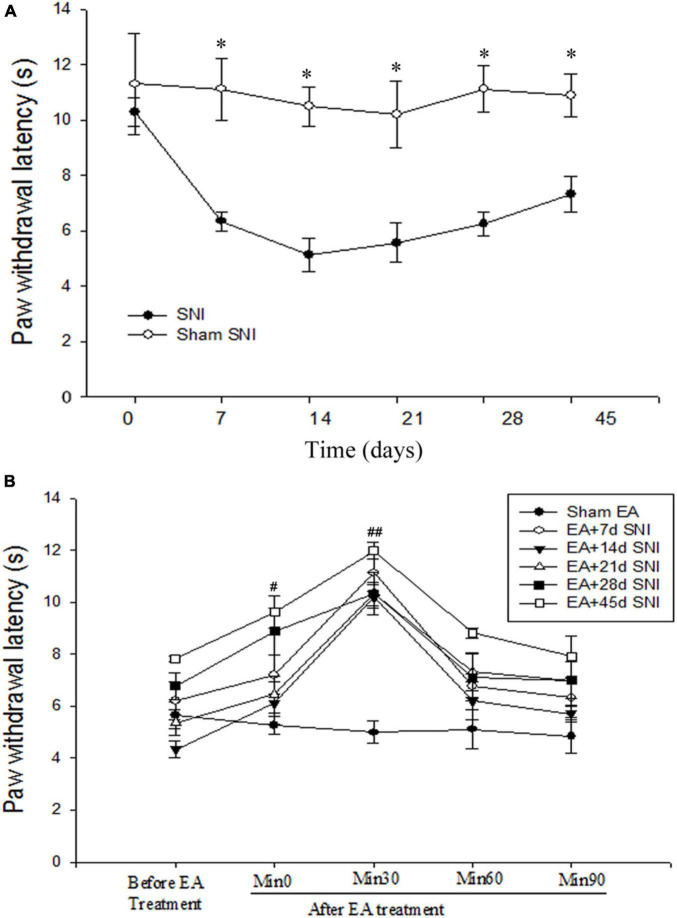
Immediate antihyperalgesic effects of EA at the different stages of spare nerve injury (SNI). **(A)** Thermal hyperalgesia during 45 days after the SNI operation. *p < 0.01. **(B)** Immediate effects of single EA on thermal hyperalgesia during the different stages of SNI. ^#^*p* < 0.05; ^##^*p* < 0.01 vs. PWL scores before EA stimulation. There were no significant differences in PWL scores before and after Sham EA (*p* > 0.05).

**FIGURE 2:**
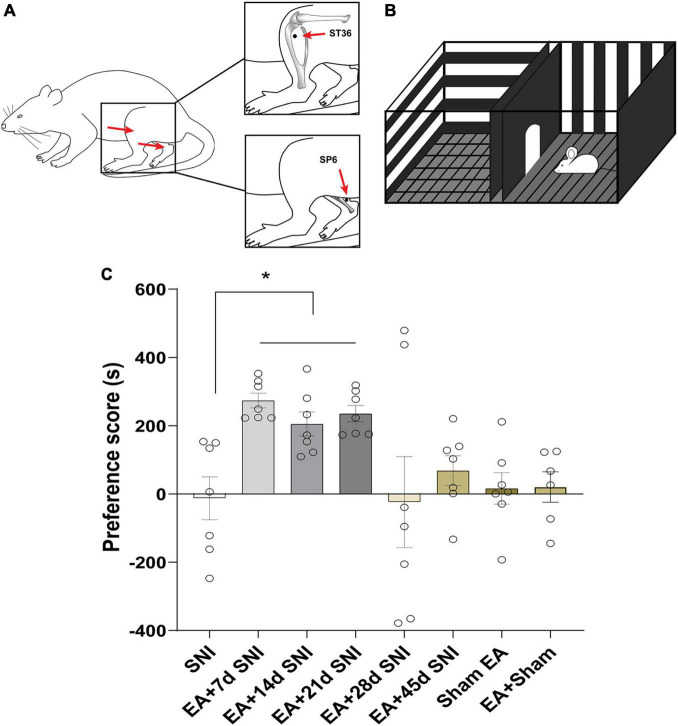
Electroacupuncture induced CPP at the early phase of chronic pain but not at the late phase. **(A)** Placement of EA stimulation. EA was performed at the acupuncture points ST36 and SP6 (solid circle). **(B)** A standard two-chamber for CPP conditioning and test. **(C)** The preference scores among the various groups. *p < 0.01 vs. SNI group.

### Behavioral Testing

#### Paw Withdrawal Latency

We measured paw withdrawal latency (PWL) in the rats by using an IITC Model 390 Paw Stimulator Analgesia Meter (IITC/Life Science Instruments, United States). We placed the rats in an inverted plastic cage. After the rats were accommodated for 30 min, a radiant heat was used to warm the hind paw of rats until they lifted their paw. We adjusted the radiant heat intensity to induce the response around 12–13 s in a normal rat. Thereafter, the PWL scores were analyzed by calculating the time from onset of radiant heat application to the paw withdrawal. The test was repeated three times independently in each rat to calculate the mean values of the PWL scores.

#### Conditional Place Preference

The CPP paradigm is shown in Figure 2B. A standard two-chamber balanced design was used to test the reward effects of pain relief induced by EA. On the preconditioning day, the rats were allowed to travel the chambers freely for 15 min, and the amount of time spent in each chamber was recorded. Next, two conditioning sessions (for EA and gentle handling) were performed for three consecutive days. Rats were treated with either EA or gentle handling for 30 min each in the morning and afternoon. We immediately placed the rats into the pairing chamber and confined them to the chamber for 30 min after administration of 30 min EA or gentle handling. For every conditioned animal, EA and gentle handling were carried out alternatively in the morning and afternoon sessions. The morning and afternoon EA/gentle handling were conducted at least 4 h apart. A preference test was conducted 3 days after the conditioning. The rats were allowed to freely access the apparatus for 15 min. The preference scores were analyzed by calculating the amount of time the animals spent in the EA-reward paired chamber minus the time they spent in the gentle handling chamber. The rats in the SNI group were conditioned in each chamber without any specific treatment, while the Sham EA group was conditioned with Sham EA treatment.

#### Protocols for Behavioral Experiment

Chronic pain differs fundamentally from acute pain in that it can potentially induce long-term plasticity in the central nervous system during the course of the disease, leading to an association of various modulatory factors to induce distinct changes in perception and behavior. Acupuncture has shown significant analgesic effects in various animal models of chronic pain (Koo et al., 2002; Zhang et al., 2014; Li et al., 2019). However, only a few studies have previously investigated the immediate effect of EA on pain at the different phases of chronic pain. Here, we tested the immediate antihyperalgesic effects of EA on the different stages of chronic pain using an SNI animal model. We randomly divided the rats into eight different groups (eight rats per group): Sham SNI, SNI, EA + 7 days SNI, EA + 14 days SNI, EA + 21 days SNI, EA + 28 days SNI, EA + 45 days SNI, and Sham EA groups. The control group did not undergo any substantial SNI modeling. The rats received EA treatment for 30 min on day 7 (EA + 7 days SNI group), day 14 (EA + 14 days SNI group), day 21 (EA + 21 days SNI group), day 28 (EA + 28 days SNI group), and day 45 (EA + 45 days SNI) after the establishment of the SNI model. Sham EA rats were subjected to the same treatment regimen but were not given any electrical stimulation. Thermal hyperalgesia was thereafter determined for 90 min after the end of the EA treatment using the PWL test. The CPP procedure induced by 4-day EA stimulation was performed on the day following the PWL test. Five Sham SNI rats subjected to the same EA regimen were as controls.

### Cannula Surgeries and Microinjections

#### Cannula Surgeries

We implanted cannulae in the LH and the NAc shell similar to those described in our previous publications (Hou et al., 2021). The rats were first anesthetized with pentobarbital sodium (50 mg/kg, i.p.) and were then mounted on a stereotaxic frame. During the surgery, the rats received isoflurane through a nosecone (1.5–2%). We then implanted guide cannulae (26 gauge, Plastics One) in the LH (−2.7 mm AP; ±1.7 mm ML; −8.6 mm DV) or the NAc (+1.8 mm AP; ±2.1 mm ML; −7.3 mm DV; 10° from Bregma). The cannula was then fixed to the skull with dental cement, and three steel screws and obturators were inserted into the cannula immediately following the surgery, with the tips extending into the intended site of injection. The rats were given systemic antiseptic solution (benzylpenicillin sodium, 60,000 units) to avoid contamination and were allowed to recover 5–7 days after the surgery.

#### Microinfusion

The rats received a bilateral injection of SB (3.0 nmol/0.3 μl) or vehicle (DMSO) in each LH or NAc shell 5 min before the CPP test. For microinjections, the obturators were first removed from the guide cannulae and 33-gauge injector needles were inserted so that the tips extended 2 mm beyond the end of the guide cannula. The drug or DMSO solutions were then infused bilaterally and delivered over a 60-s period while the rat moved freely in a plastic housing cage. An injector cannula was kept in place for 60 s after the end of the drug infusion to facilitate the diffusion into the surrounding tissues. We verified all cannula placements by microinfusion at the injection site, removing the brains under deep anesthesia, slicing them into 40 μm sections, and staining the tissues with Nissl stain for microscopic inspection. Two rats were excluded from the CPP task because of cannula misplacements. The cannula placements are shown in the behavior-associated figures.

#### Anterograde Tracing

The rats were anesthetized by administering pentobarbital sodium (50 mg/kg, i.p.) and a small hole was drilled through the skull above the LH to remove the dura. A 30-gauge needle was lowered into the right LH (−2.7 mm AP; +1.7 mm ML; −8.6 mm DV). A total of 30 nl of CTb was delivered *via* the pressure injection. Thereafter, the injection was performed over 1 min. After the injection, the needle was left in place for 15 min. CTb injections that diffused outside LH were not included in this study. The rats were allowed to recover for 7 days after the surgery before CPP conditioning and testing. To confirm the potential CTb sites in the LH, tissue sections were reacted with rabbit anti-CTb antibodies (1:500, Sigma) and then incubated for 2 h in Alexa Fluor 488 anti-rabbit (1:500, Sigma).

### Immunohistochemistry

The rats were anesthetized with 100 mg/kg pentobarbital sodium (i.p.) 90 min after CPP test. Next, rats were transcardially perfused with 200 ml saline and 250 ml of 4% paraformaldehyde in 0.1 mol/L PB. The brains were then removed and placed in a fresh fixative for an additional 4 h at 4°C. The brains were thereafter stored in 30% sucrose at 4°C for 3–5 days. The coronal sections (40 μm) of the different brain areas were cut using a cryostat at −25°C. All the sections were collected in 0.01 M phosphate-buffered saline (PBS).

### c-Fos/Orexin Double Labeling

The sections were stained for c-Fos and orexin using procedures previously described by us (Hou et al., 2021). We incubated free-floating sections in 1% bovine serum albumin for 2 h at 4°C. Thereafter, the sections were sequentially incubated at 4°C with two different primary antibodies, including anti-mouse c-Fos primary antibody (1:500; ab208942, Abcam, Cambridge, United Kingdom) and orexin polyclonal antibody (1:1,000, produced by recombinant technology for high batch-to-batch consistency; ab255294, Abcam) for 48 h. Thereafter, the sections were incubated in the goat anti-mouse IgG (1:500; ab150115, Abcam) and the goat polyclonal secondary antibody to rabbit IgG (1:500; ab150077, Abcam) for 2 h at room temperature and washed again. Finally, the brain sections were placed on clean and antifluorescence quenching slides.

### Cholera Toxin b/c-Fos Double Labeling

We performed the CTb/c-Fos double label immunohistochemistry using the same procedure as described for c-Fos/orexin double labeling. We used rabbit c-Fos primary antibody (1:500, SAB2100833, Sigma) and goat anti-CTb primary antibody (1:500, C730, Sigma) as the two primary antibodies. The donkey anti-goat Alexa Fluor 488 (1:500, SAB4600387, Sigma) and goat anti-rabbit Alexa Fluor 647 (1:500, AF647, Sigma) antibodies were used as secondary antibodies.

### Neuronal Counting

We used a Leica Laser Confocal Microscope (Leica, Germany) to analyze the various stained sections. The stereotaxic plane sections of the LH and NAc shell were identified as per the instructions in Paxinos and Watson’s atlas (George and Charles, 1986), with the numbers of positive cells per section being assessed (20×). An automatically generated 200 μm × 500 μm rectangle was placed in a fixed area of the NAc shell, LH, perifornical area (PFA), and DMN. The analysis software (Image Pro, China) was used to count the number of positive neurons per section. The numbers of the positive neurons per section for analysis were determined. A blinded observer counted the number of positive neurons on the saved image. The dorsal and ventral subregions of the NAc shell were analyzed separately. Given that there were no significant differences found in either c-Fos, CTb, or CTb/c-Fos counts between the dorsal and ventral NAc shells, the results were combined after analysis. For c-Fos/orexin labeling, % of orexin neurons that were c-Fos positive in the hypothalamus were calculated.

### Statistical Analysis

The experimental data were presented as mean ± SEM. The preference scores and immunohistochemistry data among the groups were compared *via* one-way analysis of variance (ANOVA). We analyzed the PWL scores using repeated measures ANOVA (time as a within-subject factor and group as a between-subject factor). When significance was found using ANOVA procedures, *post hoc* analysis was carried out using Fisher’s LSD test. *p* < 0.05 was the threshold of significance for these analyses.

## Results

### The Immediate Antihyperalgesic Effect of Electroacupuncture During the Different Stages of Chronic Pain

As shown in Figure 1A, the PWL scores were found to be significantly reduced immediately after SNI modeling in comparison with the Sham SNI group and were maintained for at least 45 days after SNI. The immediate antihyperalgesic effect of EA was assessed on days 7, 14, 21, 28, and 45 after SNI. It was observed that compared with the PWL scores before EA stimulation, the scores were significantly increased 0 and 30 min after EA in EA + 7 days SNI group, EA + 14 days SNI, EA + 21 days SNI, EA + 28 days SNI, and EA + 45 days SNI groups (Figure 1B). No significant differences were observed in the PWL scores before and after Sham EA (*p* > 0.05). This indicated that EA produced a significant immediate antihyperalgesic effect at different stages of chronic pain (Figure 1B). The PWL scores were observed to be increased immediately after EA stimulation, reaching maximal levels within 30 min and declining to a minimum at 90 min after EA stimulation in all groups except the Sham EA group (Figure 1B).

### Electroacupuncture Induced Conditioned Place Preference at the Early Phase of Chronic Pain but Not During the Late Phase

In the CPP analyses, we found significant differences in the CPP scores among the seven different groups [*F*(7,47) = 3.84, *p* < 0.01]. As shown in Figure 2, the EA + 7 days SNI, EA + 14 days SNI, and EA + 21 days SNI rats exhibited marked preferences for the EA-paired chamber compared with the SNI group (*p* < 0.05). However, no significant differences were found in the CPP scores among the SNI, EA + 28 days SNI, EA + 45 days SNI, and Sham EA groups (*p* > 0.05). It was observed that EA did not induce CPP in the Sham-operated animals compared with the SNI group (*p* > 0.05), indicating that EA did not induce a reward in the absence of pain. These findings clearly indicated that EA induced the reward effects of pain relief at the early phase of chronic pain but not at the late phase.

### Conditioned Place Preference Induced by Electroacupuncture Activated c-Fos-Positive Orexin Neurons in the Lateral Hypothalamus

**FIGURE 3:**
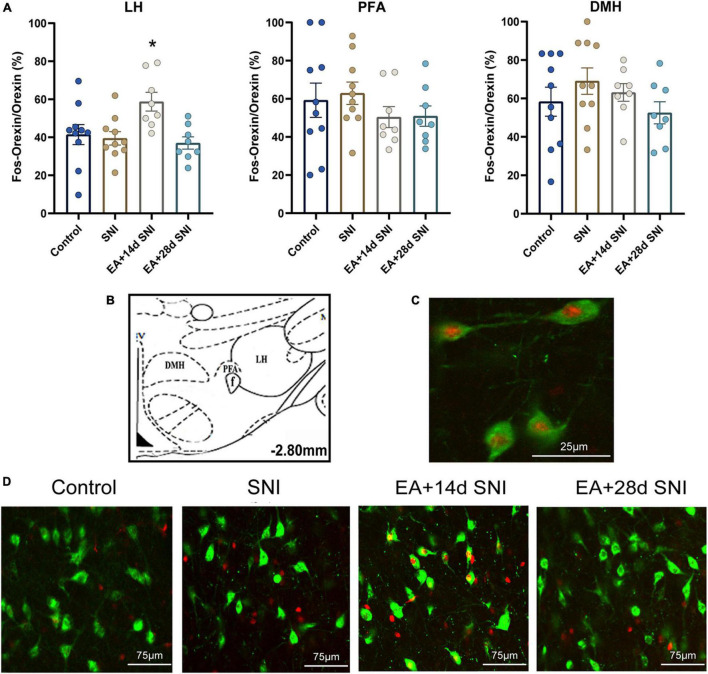
The reward effects of pain relief induced by EA-induced activation of the c-Fos-positive orexin neurons in the LH. **(A)** The percentages of orexin neurons that were observed to be c-Fos positive in the LH, PFA and DMH. *p < 0.01 vs. SNI group, control group, and EA + 28 days SNI. **(B)** Schematic coronal section through the hypothalamic subregions where stained nuclei were counted. **(C)** Double-labeled orexin (green) + c-Fos-positive (red) neurons (×40) in the LH. **(D)** Representative photomicrograph showing orexin/c-Fos immunoreactivity in the LH. Scale bar, 75 μm.

For this experiment, 1.5 h after the CPP test, 4 or 5 rats per group selected randomly from the control, SNI, and EA + 14 days SNI groups as well as the EA + 28 days SNI group and were deeply anesthetized for immunohistochemistry. We thereafter measured the total number of orexin neurons in the LH, PFA, and dorsomedial hypothalamus (DMH) expressing c-Fos protein by using double immunofluorescent labeling of c-Fos protein and orexin-A. As shown in Figure 3, significant differences in the percentages of orexin neurons that were c-Fos positive in the LH were detected among the four groups [*F*(3,32) = 4.61; *p* = 0.009]. EA stimulation on day 14 after SNI modeling increased the numbers of c-Fos-positive orexin neurons in the LH compared with the control group, the SNI group, and the EA + 28 days SNI group (*p* < 0.01). We did not notice any significant differences in the percentages of orexin neurons that were c-Fos positive in the LH among the control group, the SNI group, and the EA + 28 days SNI group (*p* > 0.05), indicating that EA on day 28 after SNI surgery did not activate LH orexin neurons. In addition, no significant differences were observed in c-Fos-positive orexin neurons among the different groups in the PFA [*F*(3,32) = 0.81, *p* > 0.05] and the DMH [*F*(3,32) = 1.14, *p* > 0.05].

### Microinjection of Orexin Antagonist SB334867 Into the Lateral Hypothalamus Can Significantly Ablate Electroacupuncture Induced Conditioned Place Preference in Spared Nerve Injury Rats

**FIGURE 4:**
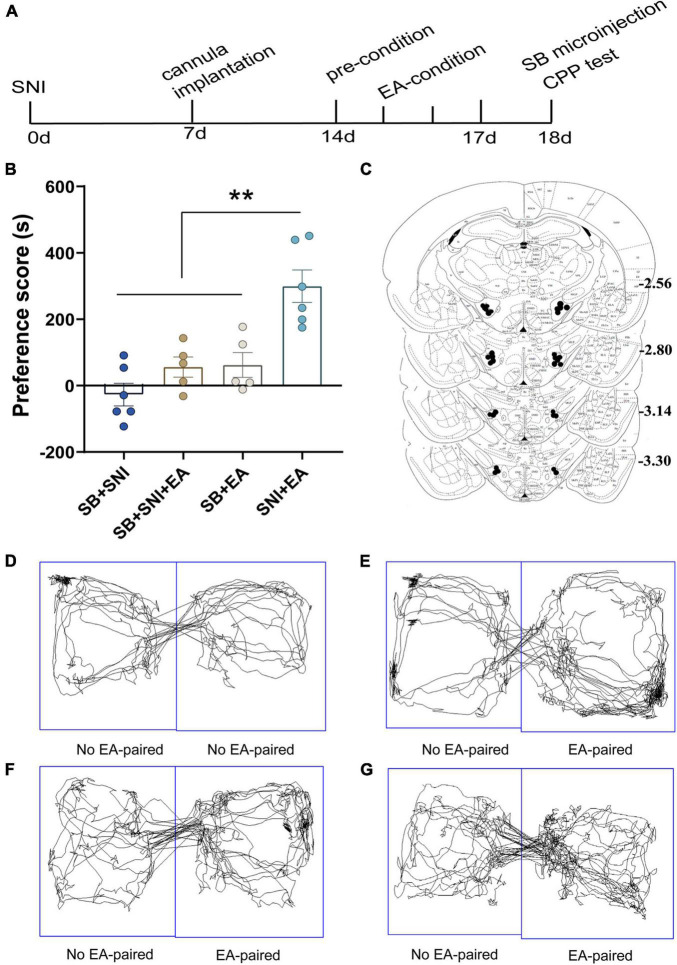
Microinjection of orexin antagonist SB334867 into the LH can significantly attenuate EA-induced CPP in SNI rats. **(A)** A timeline of SNI modeling, EA-induced CPP, SB microinjection, and CPP test. **(B)** The preference scores after CPP testing among the different groups. **p < 0.01 vs. SB + SNI group, SB + SNI + EA group, and SB + EA group. **(C)** Microinfusion cannula placements. The numbers indicate distance from Bregma in millimeters. Real-time movement traces among the groups during CPP test: **(D)** SB + SNI group; **(E)** SB + SNI + EA group; **(F)** SB + EA group; and **(G)** SNI + EA group.

To further decipher the potential effects of LH orexin neurons on the reward of pain relief induced by EA in SNI model rats, microinjection of orexin antagonist SB334867 into the LH was carried out 18 days after the establishment of the SNI model (Figure 4A). We detected significant differences in the preference scores among the different groups [*F*(3,18) = 13.77; *p* < 0.01, Figure 4]. It was found that the SNI + EA group exhibited a preference for the EA-paired chamber compared with the SB + SNI group and SB + EA group (*p* < 0.01). Moreover, LH microinjection of SB334867 (SB + SNI + EA group) significantly reduced the preference scores relative to rats in the SNI + EA group (55.8 ± 30.4s vs. 299.3 ± 49.1 s; *p* < 0.01). These observations indicated that administration of the orexin antagonist into the LH ablated EA-induced seeking behavior in SNI rats.

### The Orexinergic Neural Pathway From the Lateral Hypothalamus to the Nucleus Accumbens Could Be Activated by Electroacupuncture-Induced Reward of Pain Relief

**FIGURE 5:**
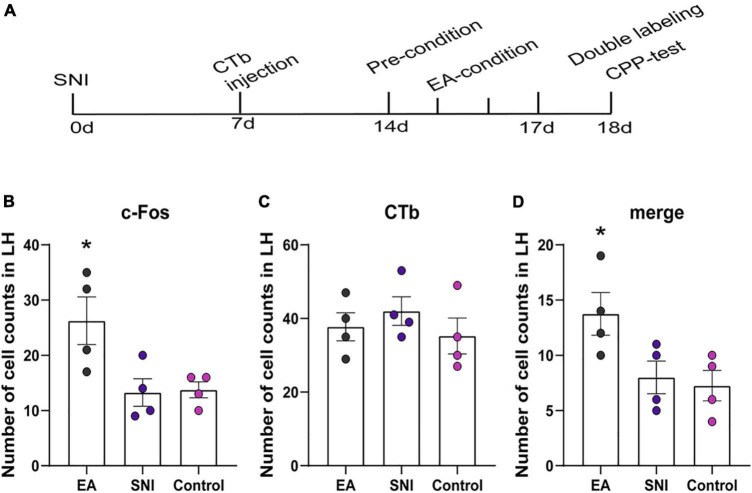
The orexinergic neural pathway from the LH to the NAc shell was activated by EA-induced CPP. **(A)** A timeline of SNI modeling, CTb microinjection, EA induced CPP conditioning and testing. **(B–D)** The number of the c-Fos positive neurons, CTb stained neurons, and double-labeled CTb/Fos neurons in the NAc shell. *p < 0.01 vs. SNI group and control group.

**FIGURE 6:**
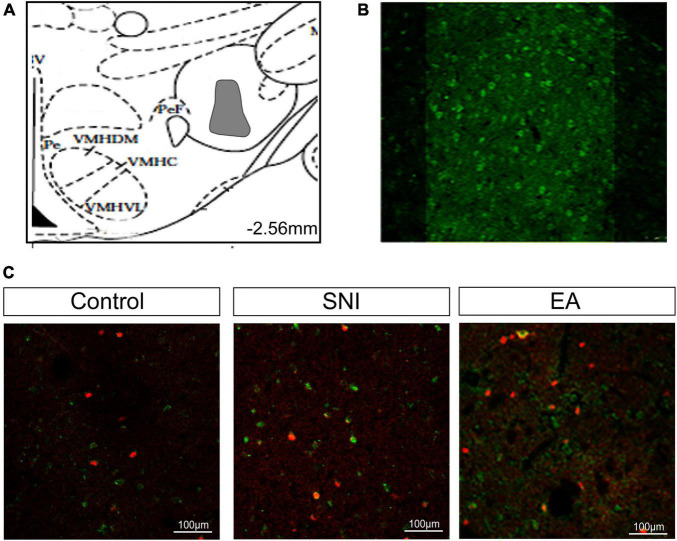
Activated afferents from the LH to the NAc shell using CTb/c-Fos double immunohistochemistry after EA induced CPP. **(A)** The maximum extent of CTb-IR (indicated gray area) in the LH. **(B)** Fluorescent photomicrographs of representative injection sites of 30 nL of CTb in the LH. **(C)** Representative immunofluorescence images for CTb/c-Fos in the NAc shell. Scale bar, 100 μm.

The NAc shell is an important target of orexin neuronal projections that has been found to be involved in orexin effects involving pain and reward. It has been suggested that the NAc shell and its interactions with LH orexin neurons might be involved in drug-, food-, and alcohol-seeking behaviors (Millan et al., 2010; Marchant et al., 2012). We next tested whether EA-induced CPP activated the afferents from the LH to NAc shell using CTb/c-Fos double-label immunohistochemistry. There were significant differences found among the different groups in the preference scores [*F*(3,18) = 13.77; *p* < 0.001, Figures 5, 6]. Moreover, the EA-treated rats still displayed higher preference scores for the EA-paired chamber compared with the SNI group (*p* < 0.05). The distribution of CTb neurons after microinjection into the LH was observed to be similar to the previous studies, including the NAc shell, the prefrontal cortex, the raphe, the locus ceruleus, and the ventral tegmental area (Hamlin et al., 2008; Petrovich et al., 2012). We primarily focused on NAc shell in the present study. Figure 6B shows a representative photomicrograph of CTb microinjection in the LH. There was significant difference found among the various groups in c-Fos expression and double-labeled CTb/Fos neurons in the NAc shell [*F*(2,9) = 6.06, *p* < 0.05; *F*(2,9) = 4.87, *p* < 0.05; respectively]. The EA groups also showed marked increases in c-Fos-positive orexin neurons in the NAc shell compared with the control and the SNI groups (Fisher’s LSD test; *p* < 0.05; Figure 6C). No significant difference was observed among the different groups in CTb neurons in the NAc shell [*F*(2,9) = 0.66, *p* > 0.05].

### Microinjection of SB334867 Into the Nucleus Accumbens Shell Ablates Electroacupuncture-Induced Conditioned Place Preference in Spared Nerve Injury Rats

**FIGURE 7:**
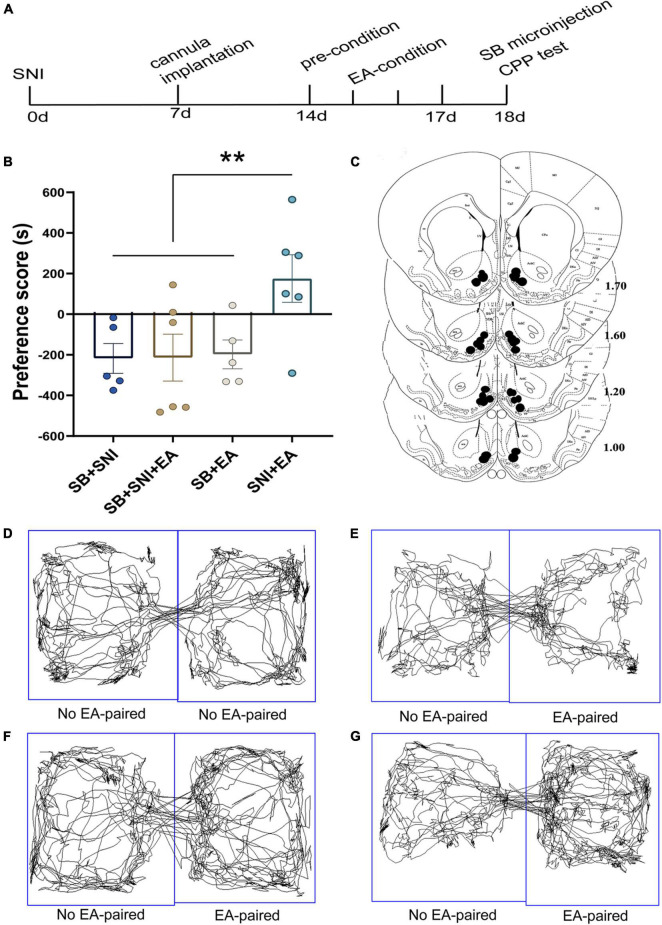
Microinjection of orexin antagonist SB334867 into the NAc can markedly ablate EA induced CPP in SNI rats. **(A)** A timeline of SNI modeling, EA-induced CPP, SB microinjection and CPP test. **(B)** The preference scores after CPP testing among the various groups. **p < 0.01 vs. SB + SNI group, SB + SNI + EA group and SB + EA group. **(C)** Microinfusion cannula placements. The numbers indicate distance from Bregma in millimeters. Real-time movement traces among the different groups during CPP test: **(D)** SB + SNI group; **(E)** SB + SNI + EA group; **(F)** SB + EA group; **(G)** SNI + EA group.

Next, we further characterized NAc shell as a critical site of orexin actions during the reward effects of pain relief induced by EA. The rats were implanted with cannulae in the NAc shell as described in the experimental techniques. The rats were given one week of recovery time after the surgery before CPP conditioning and testing. Thereafter, the rats were given an injection of SB or vehicle (DMSO) in each NAc shell 30 min prior to being placed in the CPP test (Figure 7A). Significant differences were found among the various groups in the preference scores [*F*(3,18) = 3.83; *p* = 0.03, Figure 7]. The SNI + EA group displayed a significant preference for the EA-paired chamber compared with the SNI group (*p* < 0.01). NAc shell microinjection of SB334867 significantly reduced the preference scores relative to the rats in the SNI + EA group (*p* < 0.05).

### Effects of Combined Electroacupuncture and Low-Dose Orexin-A Treatment on the Thermal Hyperalgesia and Conditioned Place Preference Scores

**FIGURE 8:**
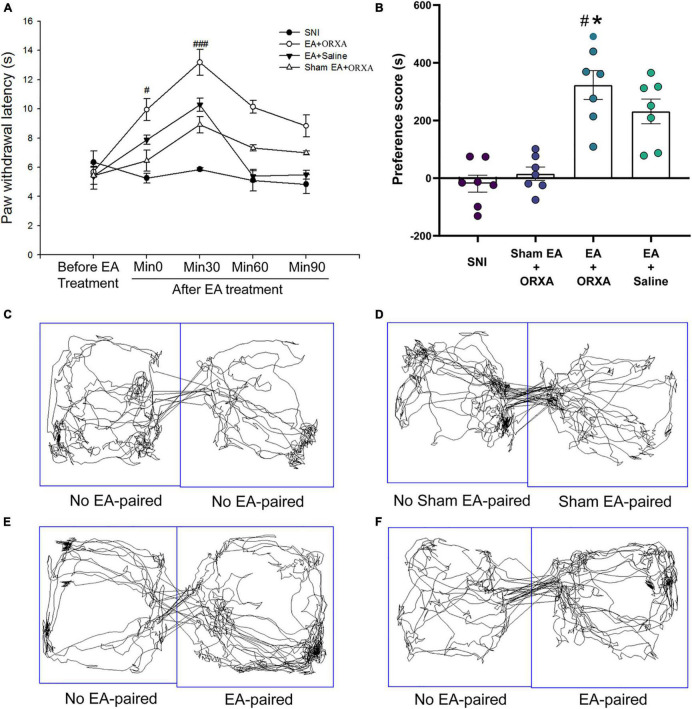
The potential effects of combinations of EA and low-dose orexin-A on the thermal hyperalgesia and CPP scores. **(A)** Immediate effects of EA and low-dose orexin-A on the thermal hyperalgesia among the various groups. ^###^*p* < 0.05 vs. SNI group, EA + Saline, and Sham EA + ORXA group. **(B)** The preference scores after CPP testing among the different groups. ^#^*p* < 0.01 vs. the SNI group; *p < 0.05 vs. the EA + saline group. Real-time movement traces during CPP test: **(C)** SNI group; **(D)** Sham EA + ORXA group; **(E)** EA + ORXA group; and **(F)** EA + saline group.

We next investigated the immediate effect of EA and low-dose orexin-A on thermal hyperalgesia and the CPP scores. According to Bingham et al.’s (2001) study, orexin-A significantly increased the latency to response in a dose-dependent manner (1, 3, 10, and 30 mg/kg) when given intravenously 5 min pretest in rats. We, therefore, selected 1 mg/kg orexin-A (ORXA, i.v. injection) for the present study. The rats were divided into the following groups (*n* = 6–8 per group): (a) SNI, (b) EA + ORXA, (c) EA + saline, (d) Sham EA + ORXA. The ANOVA analysis showed significant differences in the PWL scores among the different groups [*F*(3,38) = 8.03; *p* < 0.001, Figure 8]. The EA + ORXA, EA + saline, and Sham EA + ORXA groups displayed increased PWL scores compared with the SNI group (*p* < 0.01). However, the combination of EA and orexin-A (EA + ORXA group) showed markedly higher PWL scores than those of the EA + saline and Sham EA + ORXA groups (*p* < 0.05). We also found significant differences among the various groups in the preference scores [*F*(3,18) = 13.77, *p* < 0.001; Figure 8B]. Additionally, compared with the SNI and Sham EA + ORXA animals, EA + ORXA and EA + saline rats exhibited a preference for the EA-paired chamber compared with the SNI and Sham EA + ORXA animals (*p* < 0.05). The combination of EA and orexin-A (EA + ORXA group) was found to induce higher CPP scores compared with EA + saline group (*p* < 0.05).

## Discussion

The aim of the present study was to determine whether acupuncture induces rewards of pain relief, together with investigating whether the orexin neurons in the LH are involved in this process. Using the CPP paradigm, we found that EA was indeed able to induce the reward effects of pain relief at an early phase of chronic pain but not at a late phase, which was different from the antihyperalgesic effect of EA observed during the different stages. The EA-induced reward effects of pain relief activated the c-Fos-positive orexin neurons in the LH. Thereafter, by using CTb combined with Fos detection, we found that LH-projecting orexin neurons in the NAc shell were substantially activated during CPP induced by EA. Moreover, microinjection of the orexin-A antagonist in the LH and NAc shell blocked EA reward induction. A combination of both EA and low-dose orexin-A produced a greater antihyperalgesia effect and higher CPP scores. Taken together, these findings clearly demonstrate that orexin signaling in the LH can play a pivotal role in EA-induced reward effects of pain relief.

Pain relief can produce substantial negative reinforcement and is thus considered to be a potential reward. Similar to observations in humans, reward from pain relief can be demonstrated by the measurement of motivated behavior in different animals using various classically established models (for example, the CPP paradigm; Navratilova et al., 2012, 2015; Porreca and Navratilova, 2017). Our results showed that EA induced a significant preference for the EA paired chamber in the CPP test, thereby clearly indicating the reward of pain relief by EA in chronic pain. This finding is consistent with our clinical observations that the expectation and pleasure of pain relief could be induced by acupuncture and EA in many patients with chronic pain (data not shown here). Several previous studies related to acupuncture analgesia have largely focused on nociception (Vickers et al., 2018). However, given that acupuncture-induced reward of pain relief is clinically significant, targeting the reward mechanisms of pain relief induced by EA might offer a new prospect for a better understanding of the effects of acupuncture analgesia. Furthermore, analgesic agents can also significantly reverse evoked tactile allodynia and concomitantly produce CPP (King et al., 2009). Therefore, our data also provided conclusive evidence that EA can produce significant therapeutic effects in the treatment of chronic pain.

Chronic pain can last for weeks, months, or even years. Moreover, consistent with previous studies (Decosterd and Woolf, 2000; Boccella et al., 2018), we found that the SNI group developed thermal hyperalgesia from days 1 to 7 after surgery that lasted for at least 45 days. EA showed significant immediate antihyperalgesic effects at the different stages of chronic pain. Notably, the EA-treated rats exhibited marked preferences for the EA-paired chamber on days 7, 14, and 21 after SNI surgery (early stage). However, EA did not significantly increase the CPP scores on days 28 and 45 after SNI surgery (late stage). These findings suggested that there were at least two distinct stages (early stage and late stage), which might be involved in two different mechanisms mediating the reward effects of pain relief induced by EA. The reward effect of EA was found to be directly correlated with the time window of chronic pain. Earlier studies have shown that chronic pain can induce long-term plasticity, substantially alter brain structural features, and also affect brain functional activity (Baliki et al., 2012; Hashmi et al., 2013). It has been suggested that long-term plasticity in the brain may induce the distinct reward effects of EA during the different stages of chronic pain. In addition, the combination of EA and low-dose orexin-A on the thermal hyperalgesia and CPP scores in the present study focused only on the single and immediate antihyperalgesic effects, which might provide a novel therapeutic option for the clinical use of an EA/orexin-A combination for pain relief in patients with chronic pain. Of course, further repeated combination treatments need to be tested in future studies.

A great deal of data has indicated the potential role of hypothalamic orexin neurons in pain modulation and the reward process. Orexin-A microinjection into the posterior hypothalamus can effectively decrease the response of facial A- and C-fibers to heat and electrical stimulation (Bartsch et al., 2004). Moreover, Zhou et al. (2018) found that the orexin system facilitated both anesthesia emergence and pain control (Watanabe et al., 2005; Fakhoury et al., 2020). Significant hyperalgesia was observed in prepro-orexin knockout mice (Watanabe et al., 2005). Interestingly, the orexin system has also been reported to be involved in EA-induced analgesia (Chen et al., 2020). Acupuncture at PC6 acupuncture points significantly enhanced hypothalamic c-Fos–positive orexin neuronal numbers and induced higher orexin-A in the ventrolateral PAG in chronic constriction nerve injury and acute thermal nociception animal models (Chen et al., 2018). In the present study, we provide the first evidence that orexin system function can functionally extend beyond acupuncture analgesia to the reward effects of pain relief induced by EA. Hypothalamic neuropeptides can be mainly divided into two distinct types, namely, orexin-A and orexin-B. The selective orexin-A antagonist SB-334867 and orexin-A were used in the present study. A significant body of literature has indicated that the administration of orexin-A receptor antagonists, either systemically or locally into reward regions, attenuates a broad range of drug-seeking behaviors (Harris et al., 2005; Bentzley and Aston-Jones, 2015; Fragale et al., 2019; James et al., 2019). Similar to above results, our data clearly indicated the involvement of the orexin system in reward induced by EA analgesia. Orexin neurons can elicit their effects *via* affecting the orexin-A and orexin-B receptors (Sakurai et al., 1998; Harris and Aston-Jones, 2006). It is generally accepted that the orexin-A receptor is primarily involved in motivation and reward and the orexin-B receptor in the modulation of sleep/wake cycle and energy homeostasis (Tsujino and Sakurai, 2009; Jupp et al., 2011; Perrey and Zhang, 2020). Given that SB334867 is an orexin-A receptor antagonist, our findings suggested that the orexin-A receptor may be involved in the rewards of pain relief induced by EA. Our results also provided evidence that the LH orexin system is an essential part of the circuitry that can effectively integrate cues with EA-induced reward of pain relief. This view is consistent with the observed roles of orexin neurons in drug and food rewards (Harris et al., 2005; Moorman and Aston-Jones, 2009; Sartor and Aston-Jones, 2012). Whether orexin-B might also be involved in EA-induced rewards requires further clarification. Notably, acupuncture activates orexin neurons in several specific hypothalamus subregions, including the LH and the PFA (Chen et al., 2018). We found that EA induced a significant increase in c-Fos-positive orexin neurons only in the LH subregion, but not in the PFA and the DMH after the EA-induced preference test. This discrepancy might be possibly explained by functionally dichotomous orexin neurons present in the different hypothalamic subregions. As reviewed by Harris and Aston-Jones (2006) previously, orexins are functionally dichotomous, and orexin neurons in the LH are primarily involved in reward processing, whereas those in the PeF and DMH can effectively mediate the arousal and stress functions previously found to be associated with these peptides (Harris et al., 2005; Harris and Aston-Jones, 2006; Gao and Hermes, 2015). Orexin neurons in the LH, but not in the PeF and DMH, were noted to be stimulated by drug-conditioned contextual stimuli on the drug-free CPP test day (Harris et al., 2005). Our results are also consistent with these observations and support the view that the rewards induced by EA stimulation might be primarily associated with LH orexin cells.

**FIGURE 9:**
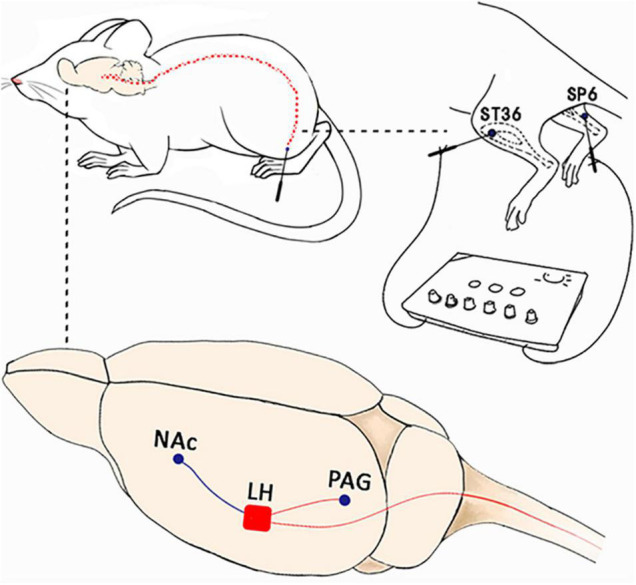
The possible mechanism involved in orexin signals in the lateral hypothalamus for acupuncture analgesia. Acupuncture signals at various acupuncture points can travel through the spinal cord to the hypothalamus. The LH orexin system is a central hub that can integrate outputs to various brain areas that can actively participate in acupuncture or EA analgesia. The projections to orexin signals in the spinal cord and PAG (descending circuits) were involved in EA-induced pain inhibition. The orexin signals in the NAc shell were found to be involved in EA-induced reward effects and inhibition of the affective component of pain.

Orexin neurons in the LH can innervate broadly in the various brain areas. Some studies have suggested that orexin might be involved in mediating the analgesic effect of EA at the spinal cord and supraspinal cord levels (Chen et al., 2020). For instance, one study showed that EA could significantly alleviate pain through modulating spinal orexin-A and its receptor interactions independent of the opioid system (Feng et al., 2012). Chen et al. (2018) focused on the PAG region and found that EA at PC6 can stimulate the release of orexin from the hypothalamus to markedly inhibiting pain responses through an endocannabinoid receptor that can reduce the inhibitory control in the PAG. We found in the present study that orexinergic neural pathway from the LH to the NAc shell could be activated by EA-induced rewards of pain relief. LH orexin neurons can heavily innervate the NAc, and the orexin-A receptor is highly expressed in this area (Trivedi et al., 1998; Marcus et al., 2001). Orexin microinjection in the NAc shell has been reported to activate NAc GABAergic and dopaminergic cells (Vittoz and Berridge, 2006; Morales-Mulia et al., 2020). In addition, orexin-A infusions into the NAc can also activate morphine-induced preferences and the injection of orexin-A antagonist into the NAc reduced the CPP for morphine (Harris et al., 2005). These results clearly indicated that orexin release in the NAc was necessary for reward effects (Taslimi et al., 2012; Sadeghzadeh et al., 2016; Assar et al., 2019). Notably, the LH-NAc pathway also plays an important role in pain modulation (Azhdari-Zarmehri et al., 2013; Yazdi et al., 2016). Chronic pain is often associated with various structural and functional abnormalities in the NAc. The offset of acute thermal stimulus can functionally increase NAc activity in healthy subjects (Baliki et al., 2010). It has been found that administration of SB334867 into the NAc dose-dependently decreased antinociception (Jahangirvand et al., 2016). A previous study has also demonstrated that the NAc appears to encode the affective value and saliency of the stimulus (Schultz, 2013; Navratilova and Porreca, 2014). In addition, fMRI studies have demonstrated that significant functional responses in the NAc were associated with acupuncture analgesia (Wang Z. et al., 2017; Yu et al., 2020b). Hence, we have suggested that the various acupuncture signals at acupuncture points can travel through the spinal cord to the hypothalamus. The LH orexin system is a central hub that can integrate outputs to several other brain areas participating in acupuncture or EA analgesia. Moreover, various projections to orexin signals in the spinal cord and PAG (descending circuits) are involved in EA-induced pain inhibition. Orexin signals in the NAc shell are also involved in EA-induced reward effects and the potential inhibition of the affective component of pain (Figure 9). The present study extends our understanding of LH orexin signaling on EA-induced rewards and provides new insights into mechanisms of acupuncture analgesia.

### Clinical Implications

Chronic pain has the potential to induce long-term plasticity in the central nervous system during the course of a disease, which can link various modulatory factors to cause a change in both perception and behavior. The findings that EA induced the preference for cues associated with EA-induced pain relief at an early phase of chronic pain but not at the late phase could help to provide novel therapeutic options in the management of patients with chronic pain. The therapeutic effects of EA may be directly correlated with the time window of chronic pain. In addition, the combination of EA and low-dose orexin-A treatments produced significantly greater antihyperalgesia effects and CPP scores. Thus, it might provide a novel therapeutic strategy for the clinical application of the EA/orexin-A combination for pain relief in patients with chronic pain.

## Data Availability Statement

The raw data supporting the conclusions of this article will be made available by the authors, without undue reservation.

## Ethics Statement

The animal study was reviewed and approved by the Shanghai University of TCM Animal Ethics Committee.

## Author Contributions

SL designed the experiment. CW, MC, CQ, XS, and SL contributed to writing and editing. CW, MC, CQ, and XQ performed the experiments and analyzed the data. XS, XQ, and SL supervised the research. All authors contributed to the article and approved the submitted version.

## Conflict of Interest

The authors declare that the research was conducted in the absence of any commercial or financial relationships that could be construed as a potential conflict of interest.

## Publisher’s Note

All claims expressed in this article are solely those of the authors and do not necessarily represent those of their affiliated organizations, or those of the publisher, the editors and the reviewers. Any product that may be evaluated in this article, or claim that may be made by its manufacturer, is not guaranteed or endorsed by the publisher.
